# Fluorine doping: a feasible solution to enhancing the conductivity of high-resistance wide bandgap Mg_0.51_Zn_0.49_O active components

**DOI:** 10.1038/srep15516

**Published:** 2015-10-22

**Authors:** Lishu Liu, Zengxia Mei, Yaonan Hou, Huili Liang, Alexander Azarov, Vishnukanthan Venkatachalapathy, Andrej Kuznetsov, Xiaolong Du

**Affiliations:** 1 Key Laboratory for Renewable Energy, National Laboratory for Condensed Matter Physics, Institute of Physics, Chinese Academy of Sciences, P.O. Box 603, Beijing 100190, China; 2 Department of Physics, University of Oslo, P.O. Box 1048 Blindern, NO-0316 Oslo, Norway

## Abstract

N-type doping of high-resistance wide bandgap semiconductors, wurtzite high-Mg-content Mg_x_Zn_1–x_O for instance, has always been a fundamental application-motivated research issue. Herein, we report a solution to enhancing the conductivity of high-resistance Mg_0.51_Zn_0.49_O active components, which has been reliably achieved by fluorine doping via radio-frequency plasma assisted molecular beam epitaxial growth. Fluorine dopants were demonstrated to be effective donors in Mg_0.51_Zn_0.49_O single crystal film having a solar-blind 4.43 eV bandgap, with an average concentration of 1.0 × 10^19^ F/cm^3^.The dramatically increased carrier concentration (2.85 × 10^17^ cm^−3^ vs ~10^14^ cm^−3^) and decreased resistivity (129 Ω · cm vs ~10^6^ Ω cm) indicate that the electrical properties of semi-insulating Mg_0.51_Zn_0.49_O film can be delicately regulated by F doping. Interestingly, two donor levels (17 meV and 74 meV) associated with F were revealed by temperature-dependent Hall measurements. A Schottky type metal-semiconductor-metal ultraviolet photodetector manifests a remarkably enhanced photocurrent, two orders of magnitude higher than that of the undoped counterpart. The responsivity is greatly enhanced from 0.34 mA/W to 52 mA/W under 10 V bias. The detectivity increases from 1.89 × 10^9^ cm Hz^1/2^/W to 3.58 × 10^10^ cm Hz^1/2^/W under 10 V bias at room temperature.These results exhibit F doping serves as a promising pathway for improving the performance of high-Mg-content Mg_x_Zn_1-x_O-based devices.

Wide bandgap oxide semiconductors such as β-Ga_2_O_3_, CeO_2_, ZnO and its alloy, Mg_x_Zn_1-x_O, have been more and more attractive for applications in ultraviolet (UV) photodetectors (PDs), photocatalyst, transparent conductive electrodes, and electronic devices etc^1^,^2^,^3^,^4^. Among them, wurtzite Mg_x_Zn_1–x_O (W-Mg_x_Zn_1-x_O) is highlighted with a combination of large, theoretically tunable bandgap (3.37–6.3 eV), low growth temperature (100–450 °C), capabilities of wet-etch processing, etc. The environment-friendly and biocompatible characteristics also make MgZnO appealing for UV device applications. Moreover, ZnO is remarkably resistant to high-energy particle irradiation, which is extremely important for UV PDs working in the outer space^5^. However, one of the biggest challenges is how to reproducibly synthesize high-quality single-phase W-Mg_x_Zn_1-x_O with high Mg content. The well-known phase segregation problem in MgZnO^6^ makes it difficult to extend the cutoff wavelength into the significant solar-blind UV spectral region. In our previous work, solar-blind 4.55 eV bandgap W-Mg_0.55_Zn_0.45_O components have been fabricated on c-sapphire by applying a unique interface engineering technique^2^,^7^. Ju *et al.*^8^ focused on synthesizing cubic MgZnO (C-MgZnO) and successfully fabricated the solar-blind UV detector, which opens up an important research direction on deep UV materials.

Another crucial issue restricting the practical use of high-Mg-content W-Mg_x_Zn_1-x_O is its notably high resistance^9^,^10^,^11^. For example, the single-crystal W-Mg_0.55_Zn_0.45_O film^7^ exhibits such high resistivity that the solar-blind UV PDs^2^,^4^ fabricated on this film demonstrate a ~5 nA photocurrent when biased at 150 V and under 254 nm UV light illumination^2^, which is insufficient for practical requirements. Tuning the conductivity is therefore specifically necessary for high-Mg-content W-Mg_x_Zn_1-x_O films and related devices. By intentionally introducing point defects—Zn interstitial, Liu *et al.*^12^ reported their interesting result on the largely decreased resistivity of MgZnO as low as 0.053 Ω · cm. Meanwhile, heterovalent cation dopants–Ga^3+^ and Al^3+^ for instance–have been added into W-Mg_x_Zn_1-x_O to create electron carriers. However, the effectiveness of these dopants as donors appears to decrease drastically as the Mg content (x) in Mg_x_Zn_1-x_O increases^13^,^14^, similar to the case of Si in Al_x_Ga_1-x_N^15^. Even more worse, such dopants like Ga might cause phase segregation in low-Mg-content W-Mg_x_Zn_1–x_O (x ≤ 0.2) films^16^. Recently, Guo *et al.*^17^ reported a quaternary alloy of Zn_0.9_Mg_0.1_OF_0.03_ to be potentially applied as transparent electrodes. There has been no report on tuning the electrical properties of high-Mg-content W-MgZnO with bandgap in solar-blind range yet. Therefore, it is worth exploring some new methods for effective n-type doping in W-Mg_x_Zn_1-x_O (x > 0.4) films in order to promote the corresponding device performance.

In this work, via comparative studies of doping with different cations and anion, a route was developed to replace O atoms with F for tuning the electrical properties of single-crystalline W-Mg_0.51_Zn_0.49_O films having a solar-blind 4.43 eV bandgap. The commercially available ZnF_2_ powder (99.995%, Alfa Aesar) chosen as the source was firstly purified and solidified in order to exclude the possibility of unwanted impurities incorporation and meet the strict requirements by radio-frequency plasma-assisted molecular beam epitaxy (rf-MBE) growth process. Our observations solidly evidence that the incorporation of F does improve the n-type conduction behavior of high-resistance W-MgZnO deep UV components. Accordingly, the UV PD fabricated with Mg_0.51_Zn_0.49_O:F epitaxial film demonstrated an enhanced photocurrent, photoresponsivity and detectivity, one or two orders of magnitude higher than that of the device fabricated on the undoped film.

## Results

**Figure 1:**
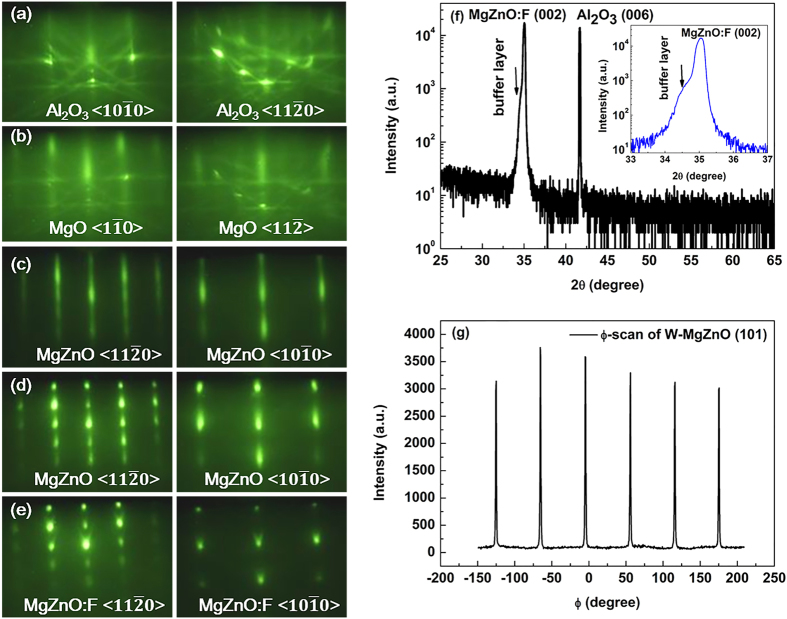
RHEED patterns with incident electron beams along the 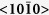 Al_2_O_3_ and 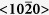 Al_2_O_3_ azimuths, respectively, obtained from Al_2_O_3_ (0001) surface (a); after growth of ultrathin MgO buffer layer at 500 °C (b); after MgZnO buffer growth at 450 °C (c); after MgZnO epitaxial growth at 450 °C (d); and after MgZnO:F epitaxial growth at 450 °C (e). XRD results of (**f**) θ*–*2θ scan of W-MgZnO:F (002) on sapphire, and (**g**) ϕ–scan of the W-MgZnO:F (101) plane. The inset shows an enlarged image of the MgZnO (002) peak, confirming the constant Mg content in the doped and undoped layers.

The samples were synthesized on sapphire (0001) substrates by rf-MBE with a base pressure of ~10^−10^ mbar. Reflection high-energy electron diffraction (RHEED) was utilized *in situ* to monitor the whole epitaxial growth process. On oxygen-terminated α-Al_2_O_3_ (0001) surface [shown in Fig. 1(a)], the ultrathin MgO (111) layer provides a good template for subsequent W-MgZnO epitaxy. It should be noted that sharp and streaky RHEED patterns of the highly strained MgO ultrathin layer overlap those of sapphire [Fig. 1(b)], indicating the achievement of an atomically flat surface inherited from the α-Al_2_O_3_ (0001) surface. The elongated, nearly streaky RHEED patterns of the low-Mg-content MgZnO quasi-homo buffer layer [Fig. 1(c)] well accommodates the large mismatch and structural discrepancy between the MgO and the high-Mg-content Mg_0.51_Zn_0.49_O epilayer [Fig. 1(d)], which becomes rough due to the much higher Mg content. It can be clearly seen that F doping does not induce any change of Mg_0.51_Zn_0.49_O structure except for the rougher surface morphology [Fig. 1(e)].

To confirm the single-crystalline wurtzite structure of the F-doped Mg_0.51_Zn_0.49_O layer, X-ray diffraction (XRD) θ*–*2θ and ϕ–scans were performed. Figure 1(f) shows the XRD θ*–*2θ curve of the F-doped sample. The peak (41.68°) is attributed to the diffraction from sapphire (006). Diffraction from W-MgZnO:F (002) planes locates at 35.04^o^ obviously shifting to a much larger angle in contrast to that of pure ZnO (34.46^o^), implying a high Mg content incorporated in the film. Importantly, the appearance of only the (002) related peak without any sign of cubic MgZnO:F confirms the single wurtzite phase, consistently with the *in situ* RHEED findings. The inset in Fig. 1(f) shows an enlarged image of the MgZnO (002) peak, confirming the constant Mg content in the doped and undoped layers. A slight asymmetry in the W-MgZnO:F (002) peak is attributed to the contribution from the low-Mg-content buffer layer underneath. In addition, Fig. 1(g) shows the ϕ–scan of the MgZnO:F (101) plane, which was carried out at χ = 60.87^o^ [the angle between (002) and (101) planes in a hexagonal system]. Six narrow and sharp peaks with equal 60^o^ intervals can be clearly observed, indicating the common sixfold symmetry of the single wurtzite crystal structure, consistent with the 60° symmetry observed in RHEED patterns.

**Figure 2:**
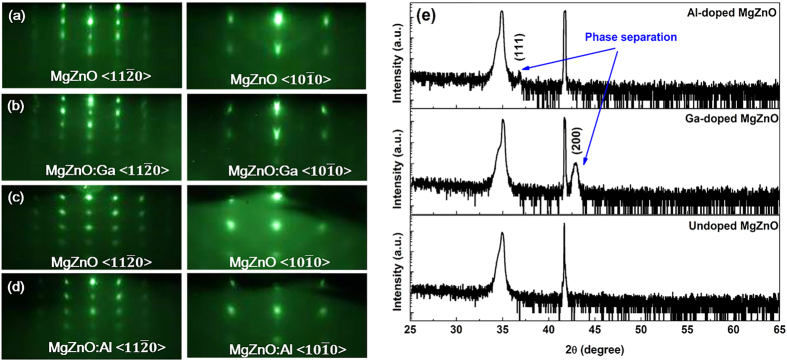
RHEED patterns obtained from MgZnO epitaxial layer before doping (a,c); after Ga or Al doping (b,d), respectively. XRD results of θ*–*2θ scans of undoped MgZnO, MgZnO:Ga and MgZnO:Al layers on sapphire (**e**).

For comparison, an intrinsic Mg_0.51_Zn_0.49_O film, Ga-doped and Al-doped alloy films were also synthesized with the same growth conditions. However, we face the difficulty in obtaining single-crystalline Mg_x_Zn_1-x_O alloy films by Ga or Al doping when increasing the Mg content x above a critical level, as other researcher encountered^14^,^16^. Following the same process as before, the undoped high-Mg-content MgZnO was synthesized, as illustrated in Fig. 2(a,c). After Ga doping, the RHEED patterns show the trend of phase segregation, indicated by the slightly twisted (02) and (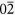) reciprocal spots [Fig. 2(b)]. As Fig. 2(e) shows, the peaks (34.96°, 34.98° and 34.89°) indicate high Mg content incorporated in these films. However, the appearance of additional peaks implies the occurrence of multiple phases after doping. Indeed, it is a challenge to dope such high-Mg-content alloy films via Ga or Al dopants. Introducing cation dopants (Ga or Al) might decrease the Mg solubility in ZnO due to the more competitive bonding between cations (Zn, Mg, Ga or Al) and anions (O). Moreover, the Ga- or Al-doped films all show huge resistance. It is therefore worth evaluating the effect of F doping on tuning the electrical properties and the device performance.

## Discussion

**Figure 3:**
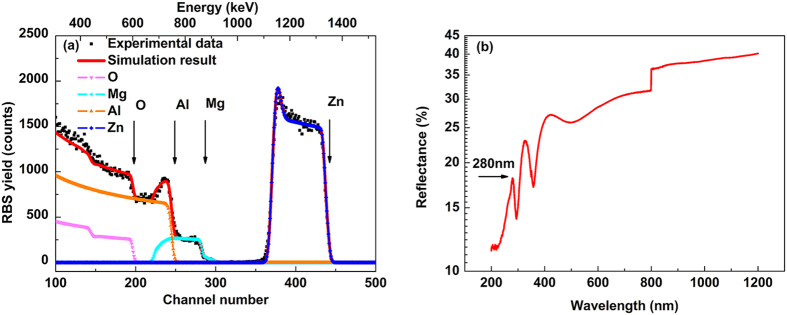
(**a**) RBS curves, and (**b**) reflectance spectrum of the same sample measured at room temperature, where the arrow indicates the bandgap position.

Figure 3(a) shows a Rutherford backscattering spectrum (RBS) taken from the F-doped sample with 2-MeV ^4^He^+^ ions backscattered into the detector at 100° relative to the incident beam direction. Arrows/labels in Fig. 3(a) indicate the channel number at which the backscattering from the corresponding atoms occurs at the surface, except for Al signal, which starts from the sapphire substrate. Note that it is difficult to distinguish F from O in RBS due to their very close mass and high background interference from O. Fitting of the experimental and simulation data [done using SIMNRA code] in Fig. 3(a) reveals the composition of the sample as Al_2_O_3_/Mg_0.36_Zn_0.64_O/Mg_0.51_Zn_0.49_O:F, consistent with the XRD measurement results.

Room-temperature reflectance spectroscopy was applied to determine the band gap of the sample. As indicated by an arrow in Fig. 3(b), the near-band edge absorption of the Mg_0.51_Zn_0.49_O:F epilayer is determined to be 280 nm, which corresponds to the optical bandgap of Mg_0.51_Zn_0.49_O:F (4.43 eV). More importantly, the bandgap of Mg_0.51_Zn_0.49_O:F is within the solar-blind range, offering the prospects of this material as active layers in the UV-C spectral region (280 nm–200 nm). The abrupt drop at 800 nm in Fig. 3(b) is induced by the exchange action of two different gratings in the testing system.

**Figure 4:**
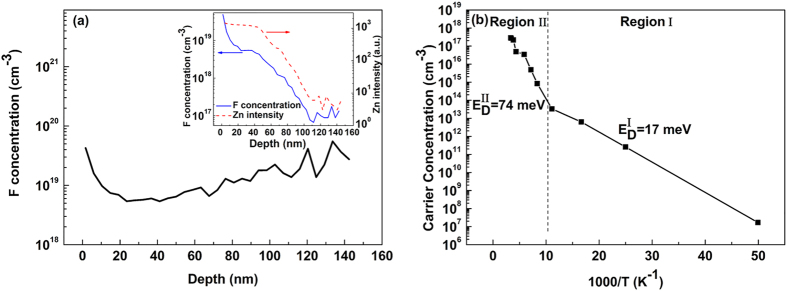
(**a**) Normalized F concentration versus depth profile of the Mg_0.51_Zn_0.49_O:F sample, and (**b**) temperature-dependent Hall results of F-doped Mg_0.51_Zn_0.49_O alloy. The inset shows the Zn intensity and the F concentration before normalization.

F concentration versus depth profile in Mg_0.51_Zn_0.49_O:F using secondary ion mass spectrometry (SIMS) is shown in Fig. 4(a). Due to the charge accumulation into the alloy film, SIMS signals, including these for Zn and F, manifest a monotonically decreasing trend, as illustrated in the inset of Fig. 4(a). Moreover, the data obtained in the range of 110–150 nm decrease a lot, close to the SIMS detection minimum limit, resulting in the huge fluctuation after normalization based on the Zn intensity [Fig. 4(a)]. It should be noted that the undoped MgZnO layer is too isolated to be measured by SIMS under the same conditions. Thus, the F-involved region is about 140 nm thick, in a good accordance with the designed thickness of the F-doped layer (~150 nm). Disregarding to the SIMS uncertainty in the vicinity of the surface as well as fluctuations in the vicinity to the inner interface, the average doping level is estimated to be 1.0 × 10^19^ F/cm^3^ [Fig. 4(a)].

In order to assess the effect of fluorine incorporation on the tuning of electrical properties, the Mg_0.51_Zn_0.49_O:F film was characterized by the temperature-dependent Hall measurement (TDH) using the van der Pauw technique in a magnetic field of 10 kG and a temperature range of 20–300 K. Figure 4(b) shows the carrier concentration (n) as the function of the reciprocal temperature, revealing two linear regions, labeled as I and II, respectively. In both regions, the electrical conductivity increases with increasing temperature. In order to determine the donor concentration (N_D_) and the activation energy (E_D_), we fitted these data by the least-squares method, assuming the charge neutrality equation for n-type semiconductor containing predominant donors and compensated acceptors:


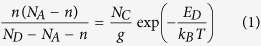


where N_A_, N_c,_ g, k_B_ and T denote the compensating acceptor concentration, the effective density of states in the conduction band, the donor degeneracy factor (~2), Boltzmann constant and absolute temperature, respectively. The impact from the semi-insulating MgZnO beneath the doped layer was reasonably neglected. The effective electron mass (m_e_*) in Mg_0.51_Zn_0.49_O:F was estimated as 0.30 m_0_ since it is reported the value changes slightly from m_e_*_(ZnO)_ = 0.29 m_0_^18^ to m_e_*_(MgO)_ = 0.30 m_0_^19^, where m_0_ is the free electron mass. As a result, the best-fitted values are as follows: for region I, 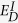 = 17 meV; and for region II, N_D_ = 8.4 × 10^18^ cm^−3^, N_A_ = 9.7 × 10^17^ cm^−3^, 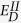 = 74 meV. In analogy to the case of GaN:Mg and ZnO:Li/Na^20^, the shallow F-donors level 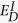 (17 meV) and the deep one 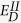 (74 meV) may be related to the lattice distortions in wurtzite Mg_0.51_Zn_0_._49_O. As we know, ZnO possesses the non-centra symmetric nature of Zn and O sub-lattices. This feature will be more pronounced with more Mg atoms incorporated, reflected by decreased c-lattice parameter confirmed by the XRD results shown in Fig. 1(f) and increased a-lattice parameter^21^. The enhanced polarization along the c-axis could affect the defect level of F in the bandgap. In wurtzite Mg_0.51_Zn_0.49_O, every substitutional fluorine atom, F_O_, will averagely form two bonds with two neighbor Mg, and another two bonds with neighbor Zn. There are two different configurations for these F_O_, i.e. one kind of F_O_ forming three bonds with two Zn and one Mg below, and one bond with one Mg up, and another kind of F_O_ forming three bonds with one Zn and two Mg below, but one bond with one Zn up (supplementary information), resulting in two different tetrahedral environments for F_O_ with different local polarization fields along the c-axis. The F-related energy level may split into two as observed by Hall measurements. The net electron concentration reaches 2.85 × 10^17^ cm^−3^ at room temperature, resulting in a resistivity as low as 129 Ω · cm and decreasing by four orders of magnitude compared to the undoped Mg_0.51_Zn_0.49_O^11^. It demonstrates that F atoms effectively act as donors by supplying free electrons when they occupy O sites in the hexagonal lattice of Mg_0.51_Zn_0.49_O.

**Figure 5:**
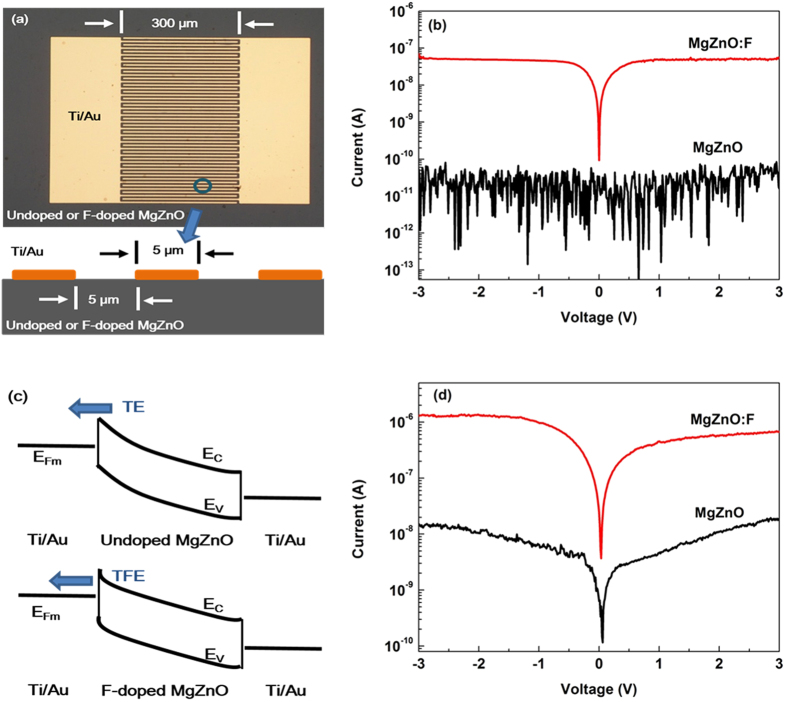
(**a**) A schematic diagram of the MSM UV PDs, (**b**) the dark current-voltage (I–V) characteristics of the two identically designed PDs using undoped and F-doped Mg_0.51_Zn_0.49_O, (**c**) energy diagram under bias, showing the dominant transport mechanism. TE = thermionic emission. TFE = thermionic-field emission, and (**d**) the current-voltage characteristics of the two PDs under 254 nm light illumination.

The impact of the enhanced conductivity on the device performance was evaluated via fabrication of two Schottky type interdigital planar metal-semiconductor-metal (MSM) UV PDs with the undoped and F-doped Mg_0.51_Zn_0.49_O, respectively. Ti (10 nm)/Au (50 nm) was deposited to form finger electrodes with 5 μm width, 300 μm length, and 5 μm gap, as illustrated in the inset of Fig. 5(a). The well-defined symmetrical rectifying behavior in Fig. 5(b,d) indicates the back-to-back Schottky contacts of the non-alloyed Ti/Au on high-Mg-content films. The dark current of F-doped Mg_0.51_Zn_0.49_O UV PD is increased by more than two orders of magnitude compared to that of the undoped one [Fig. 5(b)]. For a Schottky contact in an ideal case, if E_00_ ≈ k_B_T, the thermionic-field emission (TFE) dominates the electronic transport process, which is a combination of thermionic emission (TE) and field emission (FE). E_00_, k_B_ and T denote the characteristic energy, Boltzmann constant and absolute temperature, respectively. E_00_ is defined as:


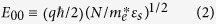


Where q, ħ, N, m_e_* and ε_s_ denote the elementary charge, the reduced Planck constant, the carrier concentration, the effective electron mass and the dielectric permittivity, respectively. The carrier concentration N is ~10^14^ cm^−3^ and 2.85 × 10^17^ cm^−3^ for the undoped and F-doped films, respectively. The dielectric permittivity in Mg_0.51_Zn_0.49_O is taken as 9.20ε_o_ by assuming a linear increase from ε_ZnO_ = 8.75ε_o_^22^ to ε_MgO_ = 9.64ε_o_^23^ with the Mg-content x in Mg_x_Zn_1-x_O. As a result, E_00_ is 0.11 meV for the undoped film, which is smaller than the thermal energy k_B_T at room temperature (26 meV). However, E_00_ for the doped one increased to a much larger value (6.0 meV) and could be to some extent comparable to the thermal energy. Therefore, the I–V curve is roughly determined by TE model and TFE model for undoped and doped cases, respectively, as illustrated in Fig. 5(c). F doping could narrow the potential barrier and result in larger tunneling currents^24^. Under 254 nm light illumination, the photocurrent of F-doped Mg_0.51_Zn_0.49_O UV PD increased by two orders of magnitude compared to that of the undoped one [Fig. 5(d)]. Thus, the photoresponsivity of the device is dramatically enhanced, although sacrificing some contrast ratio.

**Figure 6:**
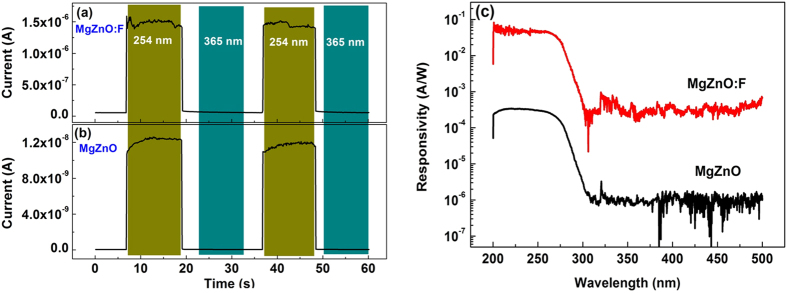
Time-dependent response of the photocurrent of undoped and F-doped MgZnO-based device (a,b) under 3 V bias. (**c**) Spectral photoresponse of the undoped and F-doped MgZnO solar-blind UV detectors at a 10 V bias.

The time dependence of photocurrent properties was studied by using periodic 254 nm and 365 nm illumination alternatively from a UV lamp [Fig. 6(a,b)]. The 10%–90% rise and decay time are less than 0.12 s for both two PDs, which is the limit of our testing system. The sharp curve [Fig. 6(a)] indicated F doping didn’t result in extra defects, especially oxygen vacancies, which could cause persistent photocurrent (PPC)^25^. Figure 6(c) shows the photoresponsivity curve of both devices at a 10 V bias. The cutoff wavelength is 278 nm and 276 nm for undoped and F-doped MgZnO-based devices, respectively, which agrees well with the optical bandgap of these two films. Note that the peak photoreponsivity was enhanced from 0.34 mA/W to 52 mA/W after F doping. (The peak at 320 nm is induced by the exchange action of two gratings in the testing system.) The noise equivalent power (NEP) can be evaluated by NEP = (4k_B_T/R_dark_ + 2qI_dark_)^1/2^
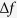
^1/2^/R, where R_dark_, I_dark_, 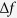
and R denote the differential resistance (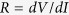) under the bias, the dark current under the bias, the electrical bandwidth and responsivity, respectively. k_B_,T and q have the same meaning as above. The NEP is determined to be 4.21 × 10^−11^ W/Hz^1/2^ and 2.22 × 10^−12^ W/Hz^1/2^ for the undoped and F-doped device, respectively, under a 10 V bias at room temperature. The detectivity (D*) can be then determined by 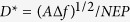, where A is the optical active area. The detectiviy D* is 1.89 × 10^9^ cm Hz^1/2^/W and 3.58 × 10^10^ cm Hz^1/2^/W for undoped and F-doped devices, respectively. The above results indicated F doping would not cause severe quality degeneration of high-Mg-content MgZnO films, but could enhance the responsivity and detectivity by 1 ~ 2 orders of magnitude. It unambiguously implies that F doping can robustly tune the conductivity of high-Mg-content W-MgZnO films in a controllable way and have a positive impact on the device performance.

To confirm the tuning effect of fluorine, the electrical properties of W-Mg_x_Zn_1-x_O:F (0≤ x ≤0.3) thin films were investigated by Hall measurement. The resistivity increased from 5.32 × 10^−3^ Ω · cm to 0.16 Ω · cm when increasing Mg content x from 0 to 0.3, with fluorine concentration of ~9.8 × 10^19^ cm^−3^ (supplementary information).

In conclusion, the conductive W-Mg_0.51_Zn_0.49_O:F thin film was prepared with a fluorine concentration of ~1.0 × 10^19^ cm^−3^, lifting the electron concentration from ~10^14^ cm^−3^ to 2.85 × 10^17^ cm^−3^. The conductivity and photoresponsivity increased by two orders of magnitude. The detectivity was enhanced from 1.89 × 10^9^ cm Hz^1/2^/W to 3.58 × 10^10^ cm Hz^1/2^/W. Two energy levels were revealed after fluorine incorporation, which is tentatively ascribed to built-in electric field discrepancy along the c-axis between two different configurations. The results indicate that F doping can dramatically modulate the electrical properties of high-Mg-content W-MgZnO and improve the UV device performance in solar-blind range, which is of crucial importance to promote the competitiveness of these active components. In addition, the purification and solidification process to ZnF_2_ powder and their unique advantages, high-purity and solid phase for example, may offer a new approach for fluorine doping attempts in other wide bandgap oxides.

## Methods

F doping into W-Mg_0.51_Zn_0.49_O film was realized with a solid anhydrous ZnF_2_ source by radio-frequency plasma-assisted molecular beam epitaxy (rf-MBE). After degreasing in acetone and ethanol, a sapphire wafer was loaded into the vacuum chamber and thermally cleaned at 750 °C, followed by exposure to active oxygen radicals at 500 °C. An ultrathin unrelaxed cubic MgO buffer layer (~1 nm) was deposited at 500 °C, providing an epitaxial template for the growth of W-MgZnO film. Further, a quasi-homo Mg_0.36_Zn_0.64_O buffer layer (~20 nm), a high-Mg-content Mg_0.51_Zn_0.49_O epilayer (~80 nm) and a fluorine-doped Mg_0.51_Zn_0.49_O epilayer (~150 nm) were subsequently grown at 450 °C, respectively. Following the same growth process, intrinsic Mg_0.51_Zn_0.49_O film, Ga-doped and Al-doped alloy films were also synthesized. The K-cell temperature for ZnF_2_, Ga and Al was set at 420 °C, 500 °C and 890 °C, respectively. More growth details can be found elsewhere^7^,^26^.

XRD was performed using Cu Kα radiation (Empyrean System). RBS system is based on 1 MeV NEC Tandem accelerator. Additional insights into the bandgap were obtained using room-temperature reflectance spectroscopy (Cary 5000 System). SIMS measurements were performed using a Cameca IMS 7 microanalyzer. The electrical properties were characterized by TDH in Lakeshore 7604 system. Semiconductor parameter analyzer (Keithley 6487) was employed for I–V characterization. The spectra response was performed using the SpectraPro-500i (Acton Research Corporation) optical system with a 75 W Xe-arc lamp combined with a 0.5 m monochromator as light source.

## Additional Information

**How to cite this article**: Liu, L. *et al.* Fluorine doping: a feasible solution to enhancing the conductivity of high-resistance wide bandgap Mg_0.51_Zn_0.49_O active components. *Sci. Rep.* **5**, 15516; doi: 10.1038/srep15516 (2015).

## Supplementary Material

Supplementary Information
