# Supplementary material for: Fluorine doping: a feasible solution to enhancing the conductivity of high-resistance wide bandgap Mg0.51Zn0.49O active components
Source: Sci Rep. 2015 Oct 22;5:15516. doi: 10.1038/srep15516 (PMC4614808; doi:10.1038/srep15516)
Supplement: Supplementary Information [file srep15516-s1.pdf]

Supplementary information

Fluorine doping: a feasible solution to enhancing the conductivity of high-resistance wide bandgap $Mg_{0.51}Zn_{0.49}O$ active components

Lishu Liu[1], Zengxia Mei[1]*, Yaonan Hou[1], Huili Liang[1], Alexander Azarov[2], Vishnukanthan Venkatachalapathy[2], Andrej Kuznetsov[2], and Xiaolong Du[1]*

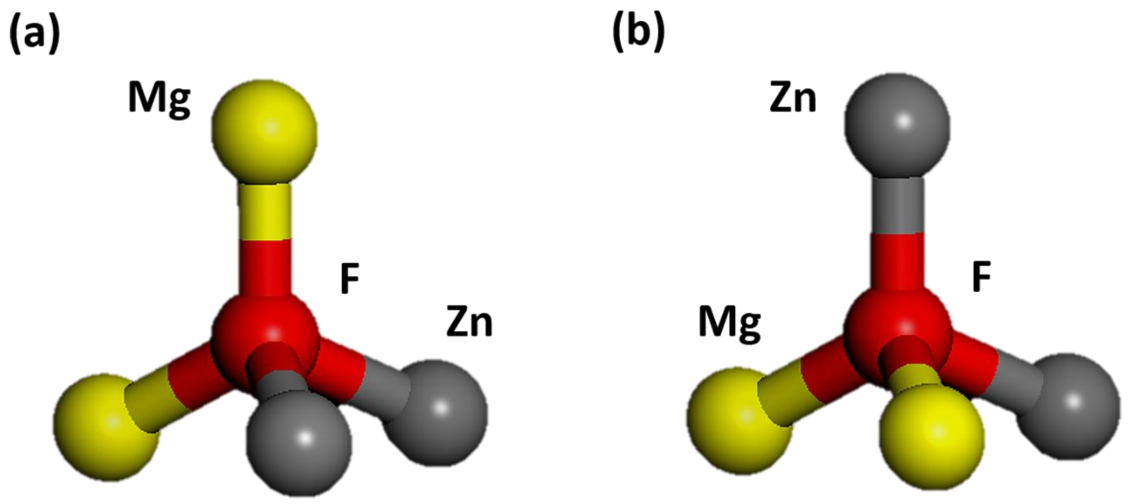

S1. Two diffirent configurations for F_O, i.e. (a) and (b), resulting in two different tetrahedral environments for F_O with different local polarization fields along the c-axis, which may be the reason for two F-related energy levels.

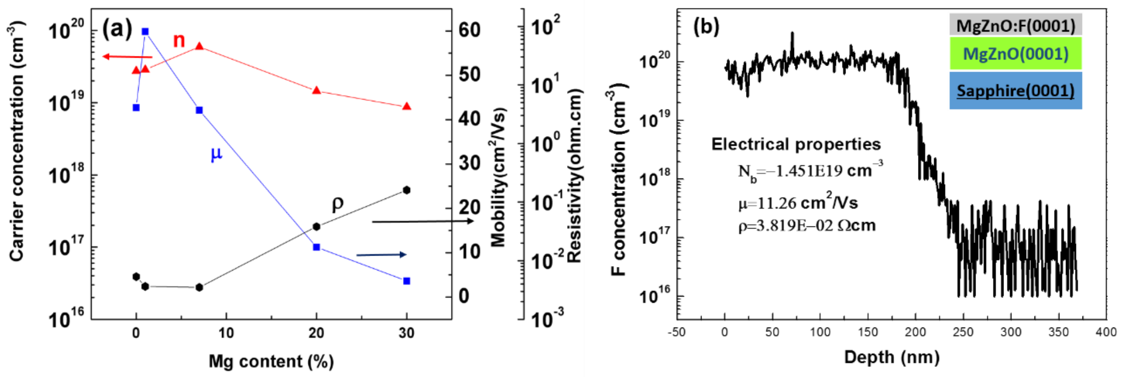

S2. (a) Electrical properties of $Mg_xZn_{1-x}O$:F with x in the range of $0 \leq x \leq 0.3$. (b) SIMS profile of $Mg_{0.2}Zn_{0.8}O$:F. The F concentration and the carrier concentration are estimated to be 9.8×10^{19} cm^{-3} and 1.45×10^{19} cm^{-3}, respectively. The inset is the schematic diagram of the sample structure.

* Authors to whom correspondence should be addressed. Electronic addresses: zxmei@iphy.ac.cn and xldu@iphy.ac.cn
